# Production of perdeuterated fucose from glyco-engineered bacteria

**DOI:** 10.1093/glycob/cwaa059

**Published:** 2020-06-27

**Authors:** Lukas Gajdos, V Trevor Forsyth, Matthew P Blakeley, Michael Haertlein, Anne Imberty, Eric Samain, Juliette M Devos

**Affiliations:** 2 Life Sciences Group, Institut Laue-Langevin, 71 Avenue des Martyrs, Grenoble 38000, France; 3 Partnership for Structural Biology (PSB), 71 Avenue des Martyrs, Grenoble 38000, France; 4 Université Grenoble Alpes, CNRS, CERMAV, Grenoble 38000, France; 5 Faculty of Natural Sciences, Keele University, Staffordshire ST5 5BG, UK; 6 Large Scale Structures Group, Institut Laue-Langevin, 71 Avenue des Martyrs, Grenoble 38000, France

**Keywords:** deuteration, engineering, *Escherichia coli*, fucose, neutron scattering

## Abstract

l-Fucose and l-fucose-containing polysaccharides, glycoproteins or glycolipids play an important role in a variety of biological processes. l-Fucose-containing glycoconjugates have been implicated in many diseases including cancer and rheumatoid arthritis. Interest in fucose and its derivatives is growing in cancer research, glyco-immunology, and the study of host–pathogen interactions. l-Fucose can be extracted from bacterial and algal polysaccharides or produced (bio)synthetically. While deuterated glucose and galactose are available, and are of high interest for metabolic studies and biophysical studies, deuterated fucose is not easily available. Here, we describe the production of perdeuterated l-fucose, using glyco-engineered *Escherichia coli* in a bioreactor with the use of a deuterium oxide-based growth medium and a deuterated carbon source. The final yield was 0.2 g L^−1^ of deuterated sugar, which was fully characterized by mass spectrometry and nuclear magnetic resonance spectroscopy. We anticipate that the perdeuterated fucose produced in this way will have numerous applications in structural biology where techniques such as NMR, solution neutron scattering and neutron crystallography are widely used. In the case of neutron macromolecular crystallography, the availability of perdeuterated fucose can be exploited in identifying the details of its interaction with protein receptors and notably the hydrogen bonding network around the carbohydrate binding site.

## Introduction

The deuteration of biomolecules by stable isotope labeling is of high interest for nuclear magnetic resonance (NMR) spectroscopy and neutron scattering studies of the structure and dynamics of biological macromolecules and is now widely used (Haertlein et al. 2016; Blakeley and Podjarny 2018). Tailor-designed deuteration can be used very effectively in studies of multicomponent systems by small-angle neutron solution scattering (Laux et al. 2008; Cuypers, Trubitsyna, et al. 2013b; Dunne et al. 2017; Josts et al. 2018; Maric et al. 2019) and neutron reflectometry (Grage et al. 2011; Hellstrand et al. 2013; Waldie et al. 2018). In Laue neutron crystallography, the use of perdeuterated protein imparts major benefits in terms of data quality and interpretation (Haupt et al. 2014; Dajnowicz et al. 2017; Yee et al. 2019; Kwon et al. 2020), and the same is true in the case of monochromatic neutron crystallography (Cuypers, Mason, et al. 2013a; Cuypers et al. 2016). Deuteration of small biomolecules has wide application in analytical methods, with growing development for the study of metabolism (Shimba et al. 1990) and in living cell imaging techniques such as Raman microscopy (Wei et al. 2013). MeV ion beam analysis techniques such as ^3^He Nuclear Reaction Analysis (NRA), where high sensitivity to deuterium was utilized to monitor the uptake of deuterated sugars in bacteria (Lowery et al. 2015) have also been deployed. More recently, it has been demonstrated that deuteration of key hydrogen atoms at positions where drugs are enzymatically metabolized for elimination has direct effects on pharmacological properties of these substances by improving their stability, and therefore their half-life, and by decreasing their toxicities (Schmidt 2017). The basis of the increased stability of these “heavy hydrogen drugs” is related to the stronger deuterium–carbon bond making the drug more resistant to enzymatic modification and elimination; deuteration of known drugs, referred to as the “deuterium switch”, resulted in a boost of patenting of labeled drugs, with several of them now in clinical trials (Timmins 2017). The first approved deuterated drug for therapeutic use was deutetrabenazine, and it has been used for the treatment of chorea in Huntington’s disease (Dean and Sung 2018).

Proteins and lipids are generally obtained in their perdeuterated form through the use of recombinant bacteria or yeasts (Meilleur et al. 2009; Haertlein et al. 2016; Moulin et al. 2018). The availability of perdeuterated and well-characterized glycans is more limited. For selective deuteration, chemical synthesis of perdeuterated monosaccharides has previously been described. Through the use of appropriate protecting groups, direct H–D exchange reactions in the presence of an activated carbon-supported platinum group catalyst were used to produce a variety of deuterated monosaccharides (Koch and Stuart 1978; Sawama et al. 2012). Using biotechnology approaches, it has been demonstrated more than 50 years ago that certain algae can be adapted to grow in 99.6% D_2_O, resulting in the large-scale production of perdeuterated glucose and mannose (Crespi et al. 1959; Chorney et al. 1960). It is only recently that cyanidin 3-*O*-glucoside was produced with a perdeuterated glucose moiety using recombinant *Escherichia coli* (Gupta et al. 2018). Polysaccharides have been produced in bacteria, in the form of perdeuterated cellulose (O'Neill et al. 2015) and perdeuterated heparin (Cress et al. 2019).

The current work focuses on l-fucose (6-deoxy-l-galactose), which is a nonclassical monosaccharide with an l-configuration correlated to unusual ring conformation, and a methyl group at C6. l-Fucose (Fuc) is present in all living kingdoms, from giant viruses (De Castro et al. 2013) to bacterial polysaccharides and plant cell walls. In mammals, it is a common component of glycolipids and glycoproteins (Vanhooren and Vandamme 1999; Schneider et al. 2017). Terminal fucosylation of oligosaccharides creates bioactive epitopes, such as the histo-blood group oligosaccharides (ABO and Lewis epitopes) (Painter et al. 1965). Human milk oligosaccharides are also rich in terminal fucose residues, which serves as a receptor analogue for pathogens, and a modulators of immune responses (Bode 2015). Fucose is therefore of interest as a biomarker for some cancers as well as a target for pathogen receptors (Heggelund et al. 2017).

The production of perdeuterated fucose, l-fucose-d_12_ (Fuc-d_12_), is therefore of high interest. The chemical approach has been described but is complex and time-consuming (Koch and Stuart 1978; Sawama et al. 2012). An alternative approach is based on the production of “recombinant” oligosaccharides in microorganisms, by engineering their metabolic pathways. This area of research was initiated at the end of the last century with the production of chito-oligosaccharides in *E. coli* (Samain et al. 1997). During the last decades, a large number of oligosaccharides have been produced using this approach, including human milk oligosaccharides that are of high commercial interest for improving infant formula milk (Priem et al. 2002). For example, α-2′-fucosyllactose, a major trisaccharide of human milk, has been obtained by introducing the FUT2 gene from *Helicobacter pylori* into *E. coli* (Albermann et al. 2001; Drouillard et al. 2006; Yu et al. 2018). This trisaccharide can serve as a substrate for a fucosidase. With appropriate engineering of microorganisms, fucose can be recombinantly produced, as demonstrated in *Saccharomyces cerevisiae* (Liu et al. 2018) and then in *E. coli* (Liu et al. 2019).

In this work, the production of perdeuterated Fuc-d_12_ (per-C-deuterated Fuc-d_8_ when dissolved in H_2_O) is described. Based on the previous construct for production of α-2′-fucosyllactose (Drouillard et al. 2006), the metabolic pathways of the previously described *E. coli* strain were further engineered with the addition of the appropriate hydrolase and removal of sugar transporter. After adaptation of the strains for cultivation in an appropriate deuterated medium, a large amount of perdeuterated fucose was produced and fully characterized.

## Results

### The fucose-producing strain

**Fig. 1:**
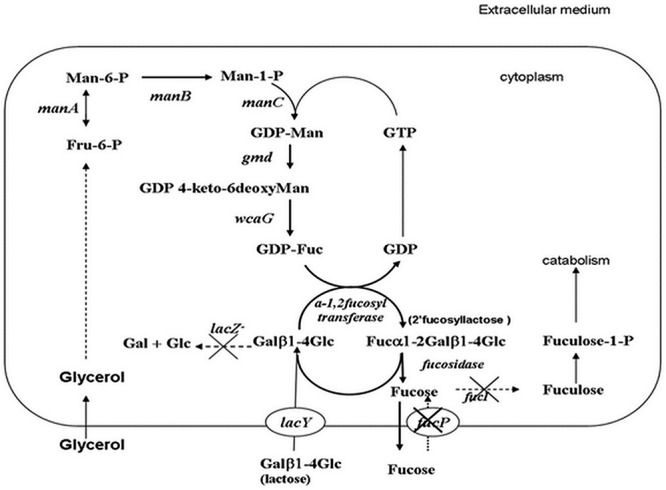
Schematic representation of the metabolic network for fucose production using the engineered *E. coli* FUC5 strain*.* Inserted genes are shown in italics and knockout genes are indicated with crosses in the diagram.

A schematic diagram of the production of l-fucose using the engineered FUC5 strain is shown in Figure 1. FUC5 is a derivative of the *E. coli* K12 strain DH1, with genetic modifications for overexpression of genes involved in GDP-fucose production, introduction of 2-fucosyltransferase gene from *H. pylori* and fucosidase gene from *Bifidobacterium bifidum* strain JCM1254, and deletion of genes involved in the metabolism of lactose. In addition, several genes have been knocked out to optimize the production of fucose: the *fucI* gene (coding for fucose isomerase) to prevent isomerization to fuculose and the *fucP* gene (fucose permease) to prevent internalization of extracellular fucose. A detailed list of the plasmids, genes and *E. coli* strains described in this work can be found in Table I.

**Table I:** Genes, plasmids and *E. coli* strains used in this study

	Description	Reference or source
Genes		
*futC*	α-1,2-fucosyltransferase from *Helicobacter pylori* strain 26695	GenBank KY499613
*afcA*	α-1,2-fucosidase from *Bifidobacterium bifidum* strain JCM1254	GenBank AY303700
*gmd*, *wcaG*, *manC*, *manB*	*E. coli* genes coding GDP-Man dehydratase, fucose synthase, GDP-Man pyrophosphorylase and phosphomannomutase, respectively	GenBank U38473.1
Plasmids		
pEXT20-futC	pEXT20 carrying *futC*	Drouillard et al. (2006)
pBP-futC	Derived from pEX20-futC	This study
pBBRGAB	pBBR1MCS-3 derivative carrying *gmd*, *wcaG*, *manC* and *manB*	Dumon et al. (2006)
pSU-fucase	pSU2718 derivative carrying active α-1,2-fucosidase domain of *afcA* from *Bifidobacterium bifidum*	This study
Strains		
DC	DH1 *lacZ lacA*	Dumon et al. (2006)
FUC	DC *fucI fucP*	This study
FUC5	FUC (pBP-futC, pBBRGAB, pSU-fucase)	This study

### Adaptation to D_2_O and batch fermentation

**Fig. 2:**
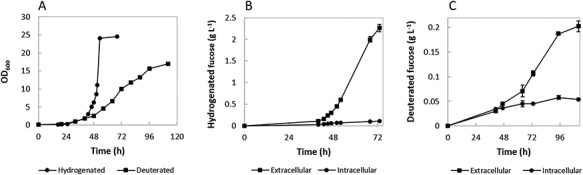
(**A**) Growth curves of hydrogenated and deuterated *E. coli* FUC5 in high cell-density cultures. The optical density was measured at 600 nm using a spectrophotometer and was plotted against time (h). (**B**, **C**) Production of hydrogenated (**B**) and deuterated (**C**) fucose using the genetically modified *E. coli* FUC5. The concentrations of extracellular and intracellular fucose were measured spectrophotometrically using the K-Fucose kit (Megazyme). Symbols represent a mean value of two independent measurements. Error bars indicate the standard deviation (NB some of the error bars are smaller than the symbol size used in the panels).

In order to compare fucose production in different conditions, the FUC5 strain was grown in both unlabeled and deuterated minimal medium supplemented with hydrogenated glycerol and deuterated glycerol-d_8_, respectively. Its growth was stepwise adapted to deuterated media as previously described (Haertlein et al. 2016). Both hydrogenated and deuterated fucose were produced in simple batch fermentations. The hydrogenated culture showed a higher growth rate in the exponential phase, reaching a maximum OD_600_ of 26, 73 h after inoculation. The deuterated culture showed a similar lag phase profile but slower growth in the exponential phase with the highest OD_600_ being 17, 112 h after the start of the fermentation (Figure 2A).

The bacterial growth and the deuterated fucose concentration showed a cell-density-associated production pattern (Figure 2). A maximum Fuc-d_12_ concentration of 0.20 g L^−1^ was obtained by the end of the fermentation process. The yield of fucose produced per gram of carbon source in the growth medium was 10-fold lower for the deuterated culture (0.2 vs. 2.1 g L^−1^, see Figure 2B and C) when supplied with the same quantity of carbon source, i.e., 30 g of glycerol used per liter of growth medium as indicated in the experimental section. Analysis by thin layer chromatography (TLC) showed gradually increasing concentrations of intracellular lactose transported from the medium to the cells by lactose permease. The TLC also revealed the increase in concentration of extracellular fucose over time (Figures S1 and S2).

### Optimization of purification method

**Fig. 3:**
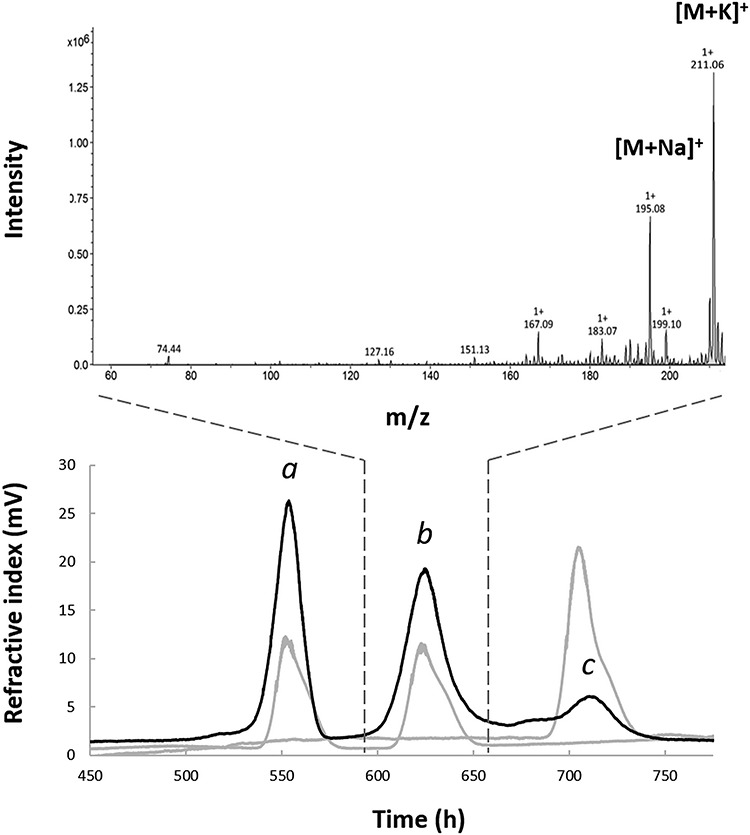
(**Top**) Mass spectrometry and (**Bottom**) chromatography analyses of per‐C‐deuterated fucose produced. Analyses were carried out in H_2_O and thus correspond to Fuc-d_8_. Bottom: size-exclusion chromatography (SEC) profiles of the saccharides present in the extracellular fraction of the fucose-producing strain after batch fermentation in deuterated media: (a) lactose, (b) deuterated fucose and (c) isopropyl-β-d-thiogalactopyranoside (IPTG) in the black line, superimposed with a SEC profile of standard solutions in grey lines. Top: only the positive electrospray ionization (ESI) mass spectrum of the deuterated fucose (Fuc-d_8_) is displayed with sodium and potassium adduct ions at *m*/*z* 196 and 211, respectively.

Deuterated fucose was purified directly from the crude extracellular fraction. After the first ion-exchange chromatography purification step, TLC analysis showed the presence of lactose and isopropyl-β-d-thiogalactopyranoside (IPTG) that were added to the minimal medium during high cell-density culture. Further fractionation of these compounds was achieved by size-exclusion chromatography (SEC) using a TOYOPEARL HW-40 column made of hydroxylated methacrylic polymer (Figure 3, Bottom). This technique was used as a polishing step to efficiently separate lactose, fucose and IPTG, three molecules of similar sizes that could be eluted as separate single peaks. After purification, 220 mg of lyophilized Fuc-d_12_ was obtained from a culture using 1.5 L of D_2_O and 45 g of deuterated glycerol.

### Deuterated fucose characterization

The metabolic incorporation of deuterium in the fucose molecule was examined and demonstrated by electrospray ionization (ESI) mass spectrometry and quantified by ^1^H NMR. The results (Figure 3, Top) showed that the purified product dissolved in a water/methanol solution generated two major peaks corresponding to the exact masses of sodium adduct ions [M + Na]^+^ and potassium adduct ions [M + K]^+^ at *m*/*z* values of 195 and 211, respectively, confirming the presence of per-C-deuterated l-fucose (Fuc-d_8_).

**Fig. 4:**
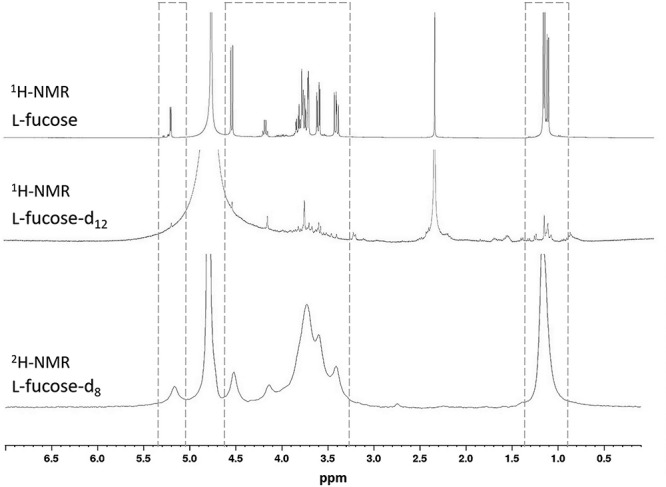
^1^H (**Top** and **Middle**) and ^2^H (**Bottom**) NMR spectra comparing the signal intensity for hydrogenated l-fucose and deuterated l-fucose. Peaks in the dashed boxes correspond to the hydrogen (^1^H spectra) and deuterium (^2^H spectrum) atoms of the fucose molecule. A signal of sodium succinate is observed at 2.4 ppm for the ^1^H spectra.

The extent of deuteration of the fucose ring and the methyl carbon was also verified by ^1^H and ^2^H NMR. Proton NMR spectra were measured on samples of commercially available hydrogenated l-fucose and on the deuterated l-fucose produced in this work, both solubilized in D_2_O. The deuterium NMR spectrum was measured from the deuterated fucose dissolved in water. The ^1^H spectrum of the purified compound exhibited the loss of intensity of all proton signals compared to the non-labeled standard (Figure 4). Moreover, the deuterium signals observed in the ^2^H spectrum of the perdeuterated product demonstrated that deuterium replacement has taken place successfully for all of the carbon atoms of the fucose molecule.

## Discussion

In this study, glyco-engineered *E. coli* has been used to produce deuterated fucose from deuterated glycerol and D_2_O via biosynthetic pathways. For the production of deuterated fucose-d_12_, deuterated glycerol-d_8_ was used as the sole carbon source. Even though glucose is a preferred carbon source for *E. coli*, several studies showed that engineered *E. coli* strains producing fucosyllactose and fucose give higher titers of fucose when grown on glycerol (Jung et al. 2019; Liu et al. 2019). Compared with the hydrogenated culture, the perdeuterated high cell-density culture grew more slowly in the exponential phase and gave lower titers of fucose. Similar behavior has been observed in deuteration of organic molecules using engineered organisms (Meilleur et al. 2009; Moulin et al. 2018).

The engineered FUC5 strain produced 0.2 g L^−1^ of deuterated fucose from a limited amount of glycerol in a simple batch fermentation. The fully deuterated fucose was characterized by mass spectrometry, proton and deuterium NMR, confirming full deuteration of the final product. It should be noted that the final yield of both the hydrogenated and the perdeuterated monosaccharides in this study was strongly dependent on the amount of glycerol used during the fermentation process. Due to the cost of the deuterated carbon source, this amount was limited to 45 g in both the H- and the D- studies. Under optimal hydrogenated conditions where the quantity of carbon source was not limited, the highest titer of fucose obtained was 20 g L^−1^ (data not shown).

Perdeuteration using engineered *E. coli* is therefore a convenient and effective method for a high-quantity production of fully deuterated simple and more complex sugars for use in neutron scattering experiments as well as in pharmacokinetic studies. In a first application, Fuc-d_12_ will be used as a ligand for fucose-specific lectins in single crystal neutron diffraction experiments.

Due to the larger and positive coherent scattering of deuterium compared to hydrogen in neutron scattering experiments, there is a better visibility of deuterium atoms vs. hydrogen. In this study, the ^1^H atoms changed for deuterium (^2^H) atoms in the fucose molecule will allow clearer visualization of crucial interactions between the sugar and bacterial lectins, as well as an optimization of the overall quality of the neutron data through the reduction of hydrogen incoherent scattering.

## Materials and methods

### Strains and plasmid construction

The fucose-producing strain FUC5 was obtained by transforming the host strain FUC with the three following plasmids: pBP-futC which contains the α-1,2-fucosyltransferase gene *futC* from *H. pylori* strain 26695; pBBRGAB which contains the four *E. coli gmd*, *wcaG*, *manC* and *manB* genes coding GDP-Man dehydratase, fucose synthase, GDP-Man pyrophosphorylase and phosphomannomutase, respectively; and pSU-fucase which contains the sequence of the active α-1,2-fucosidase domain of the *afcA* gene from *B. bifidum* strain JCM1254 (Katayama et al. 2004).

The host strain FUC was designed from strain DC (Dumon et al. 2006) by knocking out the genes *fucI* and *fucP* encoding fucose isomerase and fucose permease, respectively. Strain DC was a *lacA lacZ* null mutant derived from the *E. coli* K12 strain DH1 (DSM 4235). In order to knockout the *fucPI* genes, a 1.44 kb segment located between nucleotides 913 of *fucP* and 998 of *fucI* was deleted and replaced by the 5′AAGCTT sequence as follows: two DNA segments flanking the deleted sequence were amplified by PCR. The upstream 0.87 kb segment was amplified with primers 5′GGATCCGTAGATAAAGATGCAGGGCAAAGCAGAAG and 5′AAGCTTGGTTCCGGTTAAATAGTTAGCGGCAAAG, and the downstream 0.81 kb segment was amplified with primers 5′AAGCTTCGTGGCGACCGAAAACGACAG and 5′CTCGAGACCGGGCATCACATCAGGGAG. The two amplified fragments were ligated at their terminal *Hind*III restriction site and cloned together into the *BamH*I *Sal*I sites of the suicide vector pKO3. The deletion was then carried out according to the pKO3 gene replacement protocol (Link et al. 1997).

The plasmid pBP-futC was designed from plasmid pEXT20-futC (Drouillard et al. 2006) by removing the ampicillin gene using *Dra*I digestion and by inserting via blunt-end ligation a kanamycin cassette, obtained by *Pst*I digestion of the pUC4K vector.

The design of pSU-fucase plasmid was carried out as follows: a 2.73 kb DNA fragment containing the sequence of the active α-1,2-fucosidase domain of the *afcA* gene was amplified by PCR using genomic DNA of *B. bifidum* strain JCM1254 as a template and the following primers: 5′CTCGAGTAAGGAGGTAATATAATGGTCATCGCCAGTGTCGAGGACG and 5′AAGCTTAGGCGCTCGCCTTCTTCGTGATCGTGTAC. The amplified fragment was first cloned into pCR4Blunt-TOPO vector (Invitrogen) and then subcloned into the *Xho*I and *Hind*III sites of the pSU2718 expression vector to form pSU-fucase (Martinez et al. 1988).

Construction of pBBRGAB plasmid was previously described (Dumon et al. 2006).

### Production of deuterated fucose in batch fermentation

Precultures of the fucose-producing strain were first grown in LB medium. All culture media were supplemented with 15 μg mL^−1^ tetracycline, 20 μg mL^−1^ chloramphenicol, 50 μg mL^−1^ kanamycin and 4 μg mL^−1^ thiamine. All cultures were grown at 28°C with shaking at 160 rpm. The strain was adapted to deuterated minimal medium with the following composition: 5 g L^−1^ NH_4_H_2_PO_4_, 5 g L^−1^ KH_2_PO_4_, 0.5 g L^−1^ C_6_H_8_O_7_ (citric acid), 1.65 g L^−1^ KOH, 0.65 g L^−1^ NaOH, 7.5 mL trace mineral solution [13 g L^−1^ N(CH_2_CO_2_H)_3_ (nitrilotriacetic acid), 7 g L^−1^ KOH, 7.5 g L^−1^ C_6_H_5_FeO_7_ (ferric citrate), 1.3 g L^−1^ MnCl_2_.4H_2_O, 1.2 g L^−1^ ZnSO_4_.7H_2_O, 0.25 g L^−1^ H_3_BO_3_, 0.15 g L^−1^ Na_2_MoO_4_.2H_2_O, 0.21 g L^−1^ CoCl_2_. 6H_2_O, 0.13 g L^−1^ CuCl_2_.2H_2_O] and 45 g glycerol-d_8_ (Eurisotop). A single colony of *E. coli* FUC5 cells containing pBP-futC, pBBRGAB and pSU-fucase plasmids grown overnight on LB agar plates supplemented with the three antibiotics was used to inoculate 15 mL of minimal medium and was grown overnight at 28°C. The culture was then used to inoculate 15 mL of 100% D_2_O minimal medium (with deuterated glycerol-d_8_) at OD_600_ of 0.1 and grown overnight. This step was repeated five times until the doubling time for *E. coli* reached values similar to those for hydrogenated cultures. The last deuterated preculture of 200 mL was used to inoculate the 1.5 L of deuterated minimal medium in a 3 L bioreactor used for batch fermentation. The pH of the culture medium was regulated at 7.2 by addition of 4% NaOD. The temperature was maintained at 28°C. The IPTG inducer and lactose acceptor were added to a final concentration of 0.2 mM and 0.5 g L^−1^, respectively, at the inoculation time. The fermentation was stopped after consumption of the deuterated glycerol-d_8_ from the culture medium.

### Analytical methods

Cell growth was monitored by measuring turbidity at 600 nm (OD_600_) using a spectrophotometer. Culture aliquots (1 mL) were taken at different time points throughout the high cell-density culture. Samples were centrifuged and the supernatants treated as the extracellular fraction. The pellets were resuspended in 1 mL of Milli-Q water and boiled for 20 min at 100°C. After centrifugation, the supernatants were treated as the intracellular fraction. Samples were analyzed using thin layer chromatography on silica gels with an eluent composed of 1-propanol–acetic acid–water in 2:1:1 ratio. After dipping the plate in orcinol–sulphuric acid reagent, the spots were visualized by heating at 100°C.

### Purification of deuterated fucose

At the end of the fermentation, the bacterial cells were recovered by centrifugation (8000 g for 1 h at 8°C). The supernatant containing the deuterated fucose is referred to as *extracellular fraction*. The purification was performed in two steps. The first step was carried out using ion-exchange chromatography. The crude extracellular fraction was treated with a strongly acidic cation-exchange resin (Amberlite IR120 hydrogen form, Sigma-Aldrich). The resin was added progressively until the pH dropped below 3. After decantation, precipitated proteins were eliminated by centrifugation (16,000*g* for 1 h at 8°C). The supernatant was filtered and loaded onto a column with a strongly acidic cation-exchange resin (DOWEX 50WX4 hydrogen form, Sigma-Aldrich) and immediately neutralized by passing through a weakly basic anion-exchanger resin (DOWEX 66 free base, Sigma-Aldrich). The final eluate was colorless with neutral pH. The purity and amount of fucose were analyzed by thin layer chromatography. Fractions containing fucose were pooled together, sterile filtered and lyophilized. The resulting mix was further fractionated by size-exclusion chromatography using a semi-preparative TOYOPEARL® HW-40 column (Tosoh Bioscience GmbH) equipped with a refractive index detector (Knauer). The chromatography was performed at ambient temperature with water as a mobile phase and a flow rate of 120 mL h^−1^. Fractions containing pure product were pooled together and submitted to three cycles of resuspension in D_2_O and freeze-drying.

### Quantification of hydrogenated and deuterated l-fucose

Quantification of the intracellular and extracellular fucose was carried out on the cell paste and crude supernatant samples, respectively, using an enzymatic l-fucose assay kit (K-FUCOSE, Megazyme). The assay is based on the oxidation of l-fucose to l-fucono-1,5-lactone by l-fucose dehydrogenase in the presence of nicotinamide adenine dinucleotide phosphate (NADP^+^). The amount of l-fucose is stoichiometric to the production of reduced nicotinamide adenine dinucleotide phosphate (NADPH), measured by an increase of absorbance at 340 nm. The final amount of the deuterated fucose obtained was quantified by weighing the lyophilized powder after purification.

### Structural analysis of deuterated fucose

Positive and negative ion ESI mass spectrometry data were recorded on the Bruker amaZon speed ion trap mass spectrometer. A sample of deuterated fucose was solubilized in D_2_O to a final concentration of 50 μM.

The proton and deuterium NMR spectra were recorded with a Bruker Avance™ III NMR spectrometer operating at a frequency of 400.13 MHz for ^1^H and 61.42 MHz for ^2^H. For the ^1^H NMR experiments, samples of hydrogenated and deuterated fucose were solubilized in D_2_O with 1.82 g L^−1^ sodium succinate as a standard. Residual signal of the solvent was used as the internal standard: HOD at 4.8 ppm at 298 K. The ^1^H spectra were recorded at 298 K, with a 4006 Hz spectral width, 32,768 data points, 4.10 s acquisition times, 10 s relaxation delays and 16 scans.

The ^2^H NMR spectrum was measured on a sample of 7 mg of deuterated fucose dissolved in H_2_O at pH 6.7. The ^2^H spectrum was acquired at 298 K, without broadband ^2^H-decoupling, using a 90° pulse, 10,015 Hz sweep width, 3030 number of data points, 1 s acquisition time and 1024 number of scans.

## Supplementary Material

Supplementary_information_cwaa059
