# Supplementary material for: Production of perdeuterated fucose from glyco-engineered bacteria
Source: Glycobiology. 2020 Jun 27;31(2):151–8. doi: 10.1093/glycob/cwaa059 (PMC7874385; doi:10.1093/glycob/cwaa059)
Supplement: Supplementary_information_cwaa059 [file supplementary_information_cwaa059.docx]

## Supplementary Information


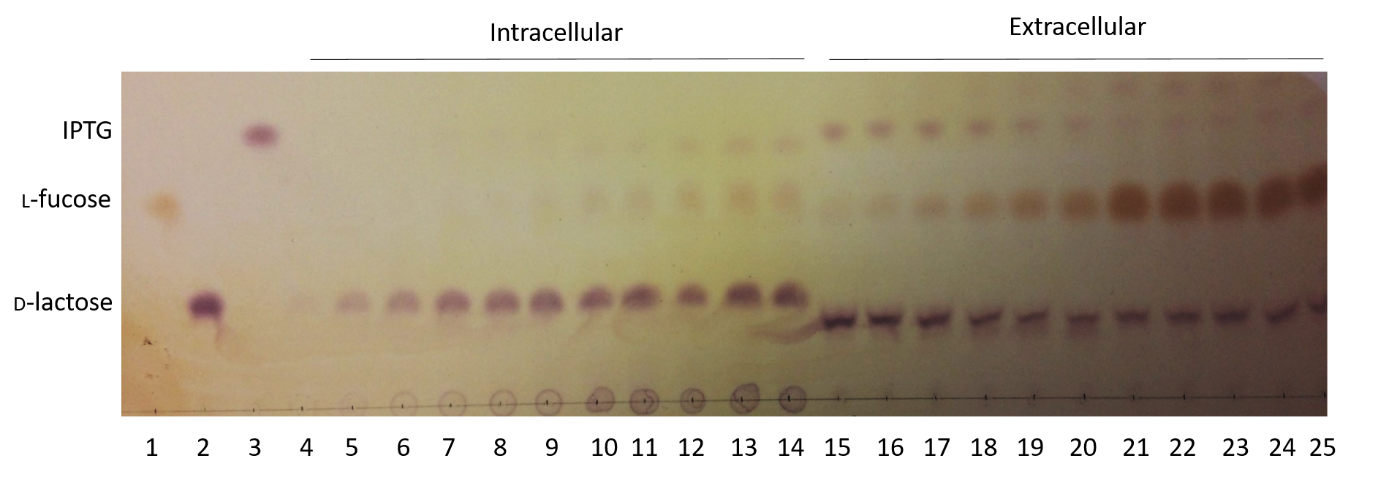


**Figure S1:** TLC analysis of intracellular and extracellular fractions of the hydrogenated high cell-density culture of *E. coli* strain FUC5 in a bioreactor. Lanes 1-3: 1 mg mL^-1^ standard solutions of L-fucose, D-lactose and IPTG respectively. Lanes 4-14: intracellular fractions withdrawn 40, 43, 45, 47, 50, 52, 64, 68, 70, 72 and 73 hours after the start of the fermentation. Lanes 15-25: extracellular fractions withdrawn 40, 43, 45, 47, 50, 52, 64, 68, 70, 72 and 73 hours after the start of the fermentation.


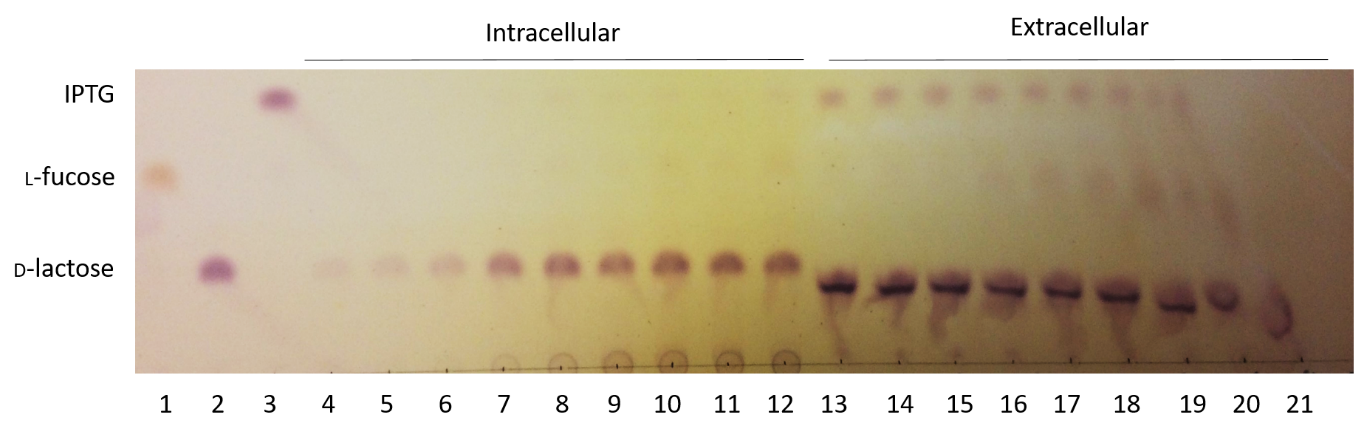


**Figure S2:** TLC analysis of intracellular and extracellular fractions of the deuterated high cell-density culture of *E. coli* strain FUC5 in a bioreactor. Lanes 1-3: 1 mg mL^-1^ standard solutions of L-fucose, D-lactose and IPTG respectively. Lanes 4-12: intracellular fractions withdrawn 40, 48, 56, 64, 72, 80, 88, 96 and 112 hours after the start of the fermentation. Lanes 13-21: extracellular fractions withdrawn 40, 48, 56, 64, 72, 80, 88, 96 and 112 hours after the start of the fermentation.
